# Long-term outcome of hypofractionated intensity-modulated radiotherapy using TomoTherapy for localized prostate cancer: A retrospective study

**DOI:** 10.1371/journal.pone.0211370

**Published:** 2019-02-26

**Authors:** Yosuke Takakusagi, Hidemasa Kawamura, Masahiko Okamoto, Takuya Kaminuma, Nobuteru Kubo, Tatsuji Mizukami, Hiro Sato, Masahiro Onishi, Nobuaki Ohtake, Tetsuo Sekihara, Takashi Nakano

**Affiliations:** 1 Department of Radiation Oncology, Gunma University Graduate School of Medicine, Showa-machi, Maebashi, Gunma, Japan; 2 Oncology Center, Hidaka Hospital, Nakao-machi, Takasaki, Gunma, Japan; 3 Department of Urology, Hidaka Hospital, Nakao-machi, Takasaki, Gunma, Japan; National Health Research Institutes, TAIWAN

## Abstract

**Background:**

Recently, the clinical outcome of prostate cancer treated by hypofractionated radiation therapy has been reported. However, there are few reports from Japan. In Hidaka Hospital, hypofractionated intensity-modulated radiotherapy (HIMRT) for prostate cancer was initiated in 2007. The purpose of this study is to analyze the long-term outcome.

**Methods:**

Ninety-two patients with localized prostate cancer treated with HIMRT at Hidaka Hospital between 2007 and 2009 were retrospectively analyzed. HIMRT was delivered using TomoTherapy. The prescription dose was 66 Gy at 95% of the PTV in 22 fractions performed 3 days a week over 7 weeks in all patients. The overall survival rate, biochemical relapse-free rate, and acute and late toxicities were evaluated.

**Results:**

The median follow-up duration was 78 (range 14–100) months. The median age at the start of the HIMRT was 72 (range 46–84) years. The disease characteristics were as follows: stage T1c, 45; T2a, 20; T2b, 5; T2c, 1; T3a, 13; T3b, 6; T4, 2; Gleason score 6, 13; 7, 44; 8, 20; 9, 15; 10, 0; pretreatment PSA ≤10 ng/mL, 42; 10 to ≤20, 27; and >20, 23. According to the D’Amico classification system, 10, 37, and 45 patients were classified as low-risk, intermediate-risk, and high-risk. The overall survival rate, the cause-specific survival rate, and the biochemical relapse-free rate at 5 years was 94.7%, 100% and 98.9%, respectively. Severe acute toxicity (grade 3 or more) was not observed. The late urinary toxicity was 52.2% in grade 0, 28.3% in grade 1, 19.6% in grade 2, and 2.2% in grade 3. The late rectal toxicity was 78.3% in grade 0, 7.6% in grade 1, 9.8% in grade 2, and 4.3% in grade 3.

**Conclusions:**

The present study demonstrated that HIMRT using TomoTherapy for prostate cancer has a favorable outcome with tolerable toxicity.

## Background

Radiotherapy is one of the definitive treatments for localized or locally advanced prostate cancer. Several studies have demonstrated dose response in prostate cancer, and the biochemical failure rates were reduced by dose escalation of radiation [1–4]. Technological improvements in radiotherapy, such as intensity-modulated radiotherapy (IMRT), can provide a higher dose to the target and spare the normal surrounding tissues [5]. Therefore, IMRT for prostate cancer has been widely used worldwide [6–8]. The standard IMRT schedule for localized or locally advanced prostate cancer ranged from 70 to 78 Gy, administered over 7 to 8 weeks [9, 10].

The α/β ratio for prostate cancer has been suggested quite low in many reports, and it is estimated between 1 and 3 [11–14]. In addition, recent publications suggest that the α/β ratio of prostate cancer was comparable or even lower to that for a late-responding normal tissue [15]. Therefore, on the basis of the α/β model for prostate cancer, hypofractionated radiotherapy, which delivers radiation dose in a smaller number of treatment with the usage of larger fraction size, would offer increased therapeutic benefit without increasing toxicity in the rectum [16]. In addition, because of the smaller number of treatment sessions, hypofractionated radiotherapy increases convenience for prostate cancer patients. Therefore, hypofractionated radiotherapy for prostate cancer has received attention recently. In fact, the clinical outcome of prostate cancer treated by hypofractionated radiotherapy has been reported, and it appeared to be comparable to conventional schedules [17–19]. However, there are fewer reports from Japan [20–22].

In Hidaka Hospital, hypofractionated intensity-modulated radiotherapy (HIMRT) using TomoTherapy for localized or locally advanced prostate cancer was initiated in 2007. The present study aimed to analyze long-term efficacy and toxicities for patients with localized prostate cancer treated with HIMRT using TomoTherapy.

## Materials and methods

### Patients

The eligibility criteria for the present study were histologically confirmed adenocarcinoma of the prostate, no evidence of pelvic lymph node involvement or distant metastasis, and a follow-up period of more than 1 year after HIMRT from April 2007 to March 2009. Ninety-two patients were retrospectively analyzed in the present study.

Written informed consent was obtained from all patients prior to treatment. All patients received a total dose of 66 Gy in 3 Gy fraction of HIMRT. Androgen deprivation therapy (ADT) was combined in 90 patients, and the median duration of ADT was 29 (range 4–123) months. The study was approved by the institutional review board of Hidaka Hospital (approval number: 218).

### Radiation therapy

In this study, all patients with prostate cancer were treated with the TomoTherapy Hi-Art system (Accuray Inc., Sunnyvale, CA, USA). It is a radiation delivery system that combines dynamic IMRT and an image-guided radiotherapy system [23, 24]. Patients were placed in the supine position. A universal fixation device (BlueBAG, EURO MEDITECH Co., LTD. Tokyo, Japan) was used to immobilize the lower legs to reduce set-up error. A planning CT scan of the pelvis was obtained at 3 mm intervals using a 16-row multi-detector CT (Aquilion LB, Toshiba Medical, Otawara, Japan). Contouring of target volumes and normal tissues was performed using the FocalSim version 4.3.1 treatment planning system (Focal Eindhoven, Netherlands). The clinical target volume (CTV) included the entire prostate and proximal seminal vesicles. In the case of T3b prostate cancer, the entire seminal vesicles were included in the CTV. The CTV was expanded in the bilateral, craniocaudal, and anterior directions with a 5 mm margin and posterior direction with 3 mm to obtain the planning target volume (PTV). The rectum and the bladder were contoured as normal tissues. Simulation CT and structure data were transferred to Hi⋅Art Planning Station (TomoTherapy Inc., Madison, WI, USA) for inverse planning.

The prescription dose was defined as the minimum dose delivered to 95% of the PTV (D95%) and was set at 66 Gy in 22 fractions. The PTV maximum dose was limited to <110% of the prescription dose. The dose limitations for rectum were aimed at no more than 60%, 35%, and 5% of the rectum volume to receive greater than 20, 33, and 54 Gy (V20 < 60%, V33 < 35%, and V54 < 17%, respectively), with a maximum dose level of 110% of the prescription dose. The dose limitations for bladder were aimed at no more than 50% and 25% of the bladder volume to receive greater than 33 and 54 Gy (V33 < 50% and V54 < 25%, respectively). Two or more radiation oncologists examined all contoured structures and treatment planning to provide consistency in radiation treatment plan.

HIMRT was given once daily, 3 days a week (Monday, Wednesday, and Friday or Tuesday, Thursday, and Saturday), resulting in a total of 22 fractions. The overall treatment duration was over 7 weeks. Image-guided radiation therapy was performed daily in all patients. The acquired CT images using mega voltage CT were superimposed onto the treatment plans. The patient’s position was adjusted according to prostate matching before each treatment.

Urologists administered androgen deprivation therapy (ADT) in 90 patients, except in two patients who were classified as low risk.

### Follow-up

A urologist and a radiation oncologist conducted patient follow-ups at 3-month intervals for the first 2 years after HIMRT and at intervals of 3–6 months thereafter. PSA was measured at each follow-up visit. Biochemical relapse was defined by Phoenix definition, that is, the nadir PSA level plus 2 ng/mL. The duration of overall survival and biochemical relapse was calculated from the start of HIMRT to the date of the death or biochemical relapse.

Toxicities were assessed according to the Common Terminology Criteria for Adverse Events ver. 4.0. Acute toxicity was defined as events occurring up to 3 months after the initiation of HIMRT and late toxicity after 3 months. The worst toxicity grade was considered the final grade of toxicity. Cumulative occurrence rates of late toxicities were estimated.

### Statistical analysis

The overall survival and biochemical relapse-free rates were estimated using the Kaplan–Meier method. Comparisons of overall survival rates and biochemical relapse-free rates were analyzed by log-rank analysis. The comparisons of patient characteristics and late toxicities were assessed using Fisher’s exact test. Statistical analysis was performed using SPSS software (version 24, Chicago, IL, USA). P < 0.05 was considered significant.

## Results

### Patient characteristics

Patient characteristics are summarized in Table 1. The median follow-up duration was 78 (range 14–100) months. The patients were classified using the D’Amico risk group classification [25]. Among 92 patients, 10 patients were classified as low-risk, 37 as intermediate-risk, and 45 as high-risk.

**Table 1:** Patient characteristics (n = 92).

Characteristics	n (%)
Age, years, median (range)	71.5 (46–84)
T stage	
1c	45 (48.9%)
2a	20 (21.7%)
2b	5 (5.4%)
2c	1 (1.1%)
3a	13 (14.1%)
3b	6 (6.5%)
4	2 (2.2%)
Pretreatment PSA, ng/ml, median (range)	10.4 (3.7–137.5)
< 10	42 (45/7%)
10 ≤ 20	27 (29.3%)
20 ≤	23 (25.0%)
Gleason score	
6	13 (14.1%)
7	44 (47.8%)
8	20 (21.7%)
9	15 (16.3%)
10	0 (0.0%)
D'Amico classification	
low	10 (10.9%)
intermediate	37 (40.2%)
high	45 (48.9%)
ADT	
none	2 (2.2%)
neoadjuvant	5 (5.4%)
neoadjuvant and adjuvant	85 (92.3%)
Radiation therapy	
66Gy in 3Gy fractions	92 (100.0%)
Diabetes mellitus	
Yes	10 (10.9%)
No	82 (89.1%)
Internal use of anticoagrants	
Yes	13 (14.1%)
No	79 (85.9%)
Follow-up duration, months, median (range)	78 (14–100)

PSA: prostate specific antigen, ADT: androgen deprivation therapy

### Overall survival and biochemical relapse-free rate

**Fig 1:**
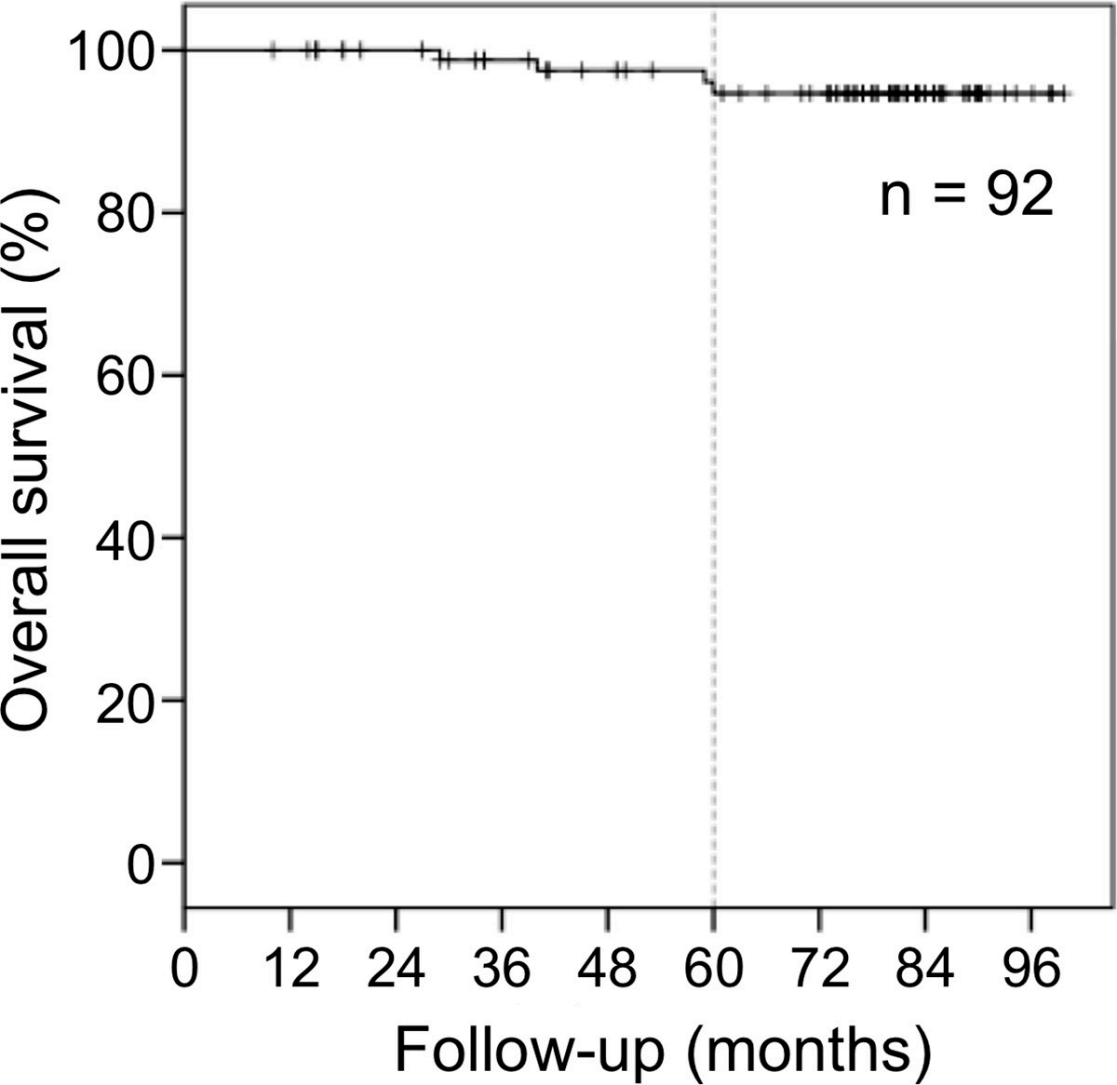
Overall survival. The overall survival rate at 5 years was 94.7%.

Survival curves are presented in Fig 1. The overall survival rate and the cause-specific survival rate at 5 years were 94.7% and 100%, respectively. Four patients died from other diseases.

**Fig 2:**
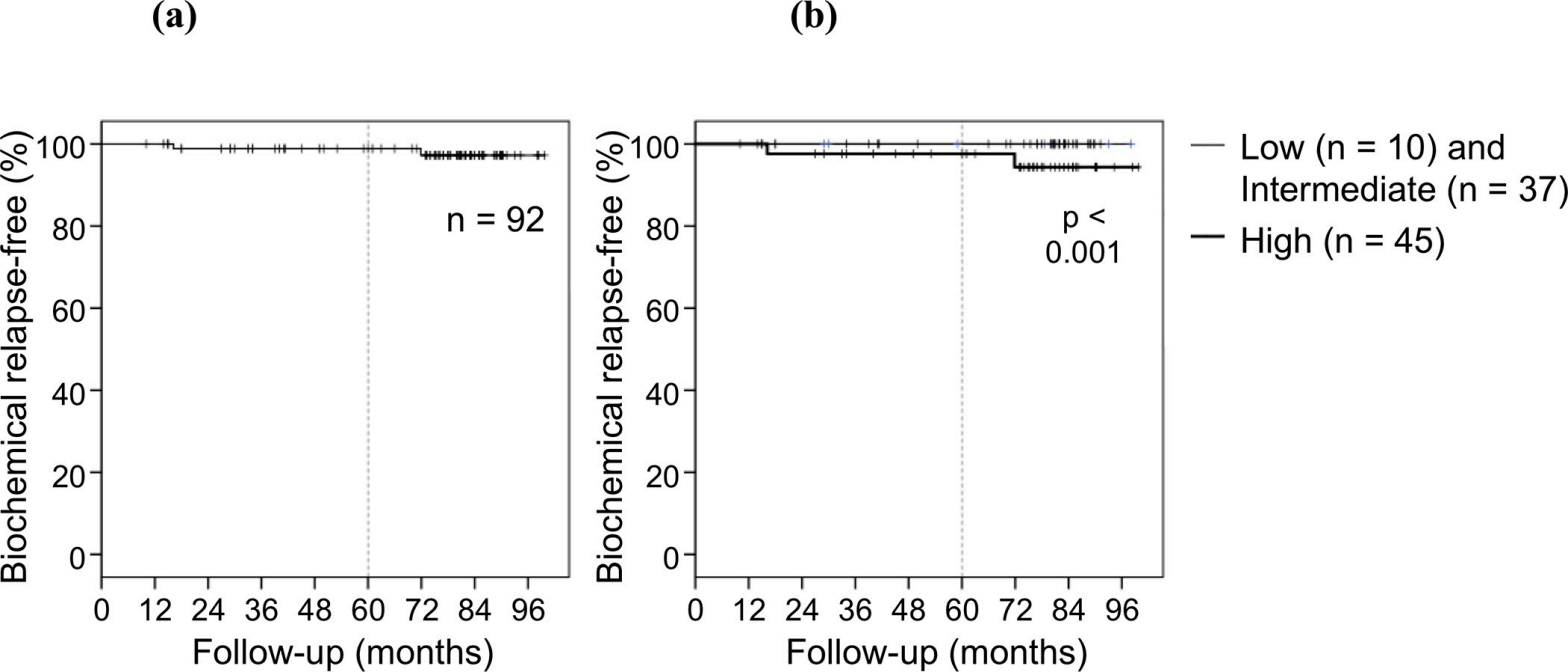
Biochemical relapse-free rate. **(a)** The biochemical relapse-free rate in all patients at 5 years was 98.9%. **(b)** The biochemical relapse-free rate in the low-, intermediate- and high-risk groups at 5 years was 100%, 100% and 97.6%, respectively. A significant difference was not observed between the low-, intermediate-, and high-risk groups (P = 0.597).

**Fig 3:**
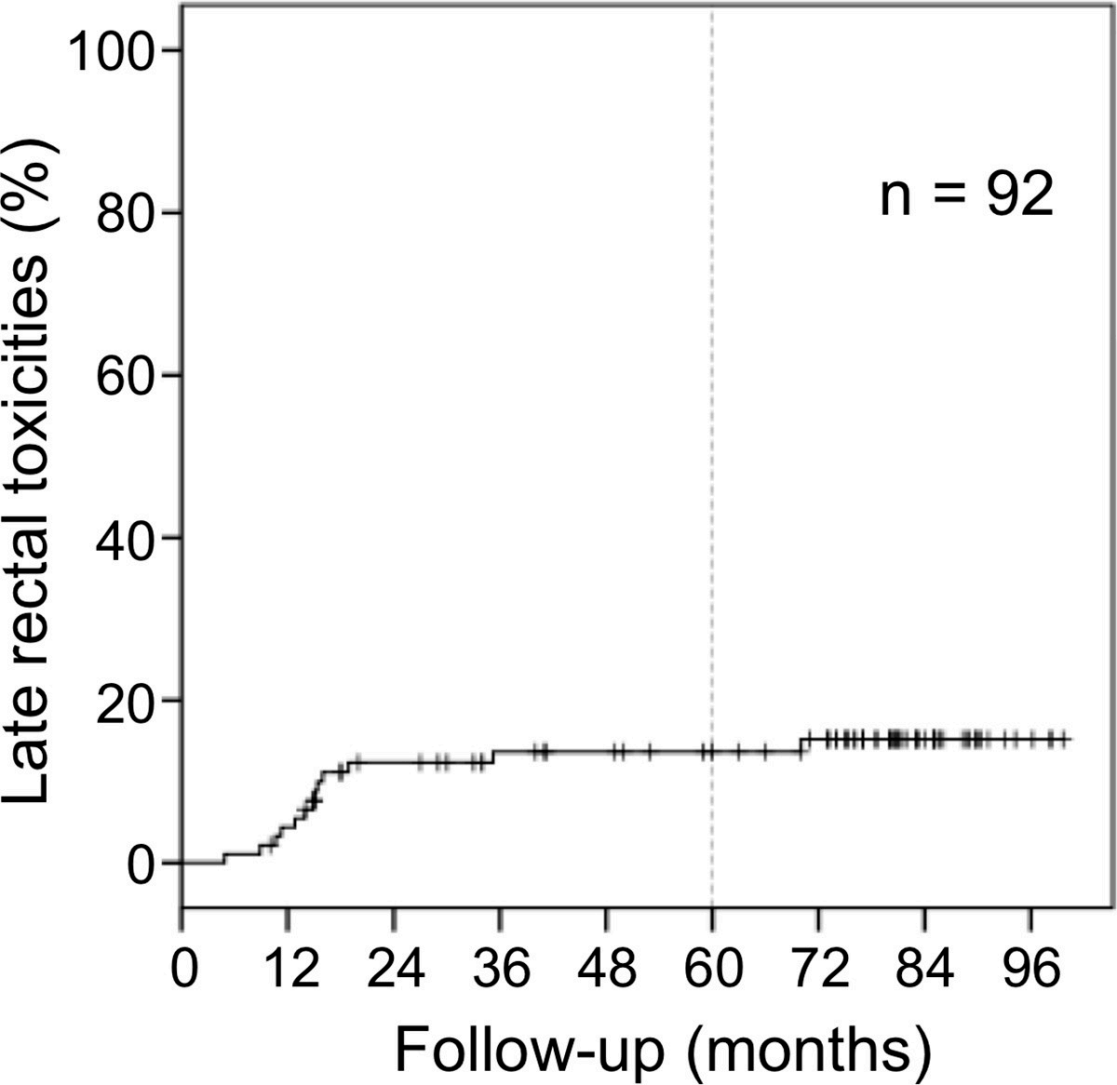
Cumulative incidence rate of grades 2 and 3 late rectal toxicities. The 5-year cumulative incidence rate of grades 2 and 3 late rectal toxicities was 13.6%. Most of the grade 2 and 3 late rectal toxicities were observed within 2 years.

The biochemical relapse-free rate at 5 years was 98.9%. The biochemical relapse-free rate is shown in Figs 2 and 3. No patient in the low- and intermediate-risk groups demonstrated biochemical relapse; however, two patient in the high-risk group demonstrated biochemical relapse. The biochemical relapse-free rate in the low-, intermediate- and high-risk groups at 5 years was 100%, 100% and 97.6%, respectively. A significant difference was not observed between the low-, intermediate-, and high-risk groups (P = 0.597). In biochemical recurrence cases, clinical recurrences were observed in two patients. One patient showed distant metastasis, and the other showed regional lymph node metastasis. No local recurrence was observed.

### Toxicities

Table 2 shows the maximal acute and late toxicities in the present study. Acute urinary toxicity developed in 48 patients (52.2%) in grade 0, 26 (28.3%) in grade 1, 18 (19.6%) in grade 2, and 0 (0.0%) in grade 3 or more. Acute rectal toxicity developed in 90 patients (97.8%) in grade 0, 1 (1.1%) in grade 1, 1 (1.1%) in grade 2, and 0 (0.0%) in grade 3 or more.

**Table 2:** Toxicities.

							n = 92
Urinary toxicities			Grade 0	Grade 1	Grade 2	Grade 3	Grade 4, 5
		acute	48 (52.2%)	26 (28.3%)	18 (19.6%)	0	0
		late	51 (55.4%)	23 (25.0%)	16 (17.4%)	2 (2.2%)	0
Rectal toxicities			Grade 0	Grade 1	Grade 2	Grade 3	Grade 4, 5
		acute	90 (97.8%)	1 (1.1%)	1 (1.1%)	0	0
		late	72 (78.3%)	7 (7.6%)	9 (9.8%)	4 (4.3%)	0

Late urinary toxicity developed in 51 patients (55.4%) in grade 0, 23 (25.0%) in grade 1, 16 (17.4%) in grade 2, and 2 (2.2%) in grade 3. No grade 4 or 5 late urinary toxicity was observed in any of the patients. Grade 3 late urinary toxicity was observed in two patients who developed massive urinary bleeding and required a blood transfusion. In one patient, urinary bleeding occurred 49 months after HIMRT initiation and the other after 54 months. Late rectal toxicity developed in 72 patients (78.3%) in grade 0, 7 (7.6%) in grade 1, 9 (9.8%) in grade 2, and 4 (4.3%) in grade 3. No grade 4 or 5 late rectal toxicity was observed in any of the patients. No significant difference was observed between grades 2 and 3 late rectal toxicities and the internal use of anticoagulants or diabetes mellitus (P = 0.064, P = 0.695, respectively). No significant difference was observed between grades 2 and 3 late rectal toxicities and other patient characteristics. Grade 3 rectal bleeding was observed at 9, 15, 15, and 70 months after initiation of HIMRT, respectively. Of these four cases, hyperbaric oxygen therapy was administered in two patients, and argon plasma coagulation through the colonoscope was administered in one patient. The cumulative incidence rate of grades 2 and 3 late rectal toxicities is shown in Fig 3. The 5-year cumulative incidence rate of grades 2 and 3 late rectal toxicities was 13.6%. Most grades 2 and 3 late rectal toxicities were observed within 2 years after the initiation of the HIMRT.

## Discussions

In the present study, we reported long-term analysis of effectiveness and toxicities for patients with localized or locally advanced prostate cancer treated with HIMRT using TomoTherapy. To our knowledge, this is the first report on long-term analysis of HIMRT using TomoTherapy for prostate cancer in Japan. The respective 5-year overall and cause-specific survival rates of 94.7% and 100% and the 5-year biochemical relapse-free rate of 98.9% appears acceptable. In addition, Grade 3 or more acute toxicities were not observed. The late urinary toxicities of grades 2 and 3 were 16 (17.4%) and 2 (2.2%) patients, respectively, and the late rectal toxicity grades were 2 in 9 (9.8%) and 3 in 4 (4.3%) patients.

Brenner and Hall first reported the low α/β ratio hypothesis of prostate cancer in 1999 [26]. The authors estimated an α/β ratio of 1.5 Gy. Following this article, investigations were conducted to estimate the α/β ratio of prostate cancer, and α/β values ranging from 1 to 3 have been reported [11–14]. Recent publications suggest that the α/β ratio of prostate cancer was comparable to that for a late-responding normal tissue or even lower because of the slow natural turnover rates in a high proportion of these tumors [27]. For the rectum, an α/β ratio >5.0 Gy is reported [28, 29]. If the α/β ratio for prostate cancer is lower than that for normal tissues, a therapeutic advantage might be gained using hypofractionated radiotherapy.

Recent developments of technologies of radiotherapy, such as IMRT or stereotactic radiotherapy, lead to delivering a higher dose to the target without increasing the toxicities in the surrounding normal tissues. Thus, there has been increasing interest in hypofractionated radiotherapy for prostate cancer. In fact, several randomized control trials of conventional versus hypofractionated radiotherapy for prostate cancer have been performed. Alwini et al. reported a randomized phase 3 non-inferiority trial of late toxicities in prostate cancer patients treated with 39 fractions of 2 Gy in 8 weeks (five fractions per week) vs. 19 fractions of 3.4 Gy in 6.5 weeks (three fractions per week) [17]. Cumulative grade 3 or worse late genitourinary toxicity was higher than that in the conventional fraction group. A significant difference was not observed in the incidence of grade 3 or worse late gastrointestinal toxicity between each group. A non-inferiority was not confirmed in this study. Catton et al. reported a randomized trial treated with 78 Gy in 39 fractions over 8 weeks vs. 60 Gy in 20 fractions over 4 weeks [18]. The biochemical relapse-free ratio at 5 years was 85% in both arms. No significant differences in grade 3 or worse late genitourinary and gastrointestinal toxicity were observed between the two arms. The CHHiP trial reported the results of the randomized non-inferiority study that compared conventional (74 Gy in 2 Gy fractions) and hypofractionated (60 Gy in 2 Gy fractions) radiotherapies for prostate cancer [19]. The 5-year free from biochemistry and/or clinical recurrence rates were 88.3% and 90.6% in the conventionally fractionated regimen group and hypofractionated regimen group, respectively, and non-inferiority was shown between these two groups. In addition, non-inferiority was shown in the late adverse event between them. Therefore, the authors concluded that the hypofractionated regimen was recommended as a new standard treatment schedule for localized prostate cancer in terms of short treatment duration and lower patient load. There were some differences in terms of the treatment schedule and patient characteristics between the CHHiP trial and the present study. Although the hypofractional regimen in CHHiP trial is carried out by treatment sessions five times a week over 4 weeks, in the present study, HIMRT was given 3 days a week over 7 weeks. T1–T3a prostate cancer with a PSA level less than 40 ng/mL was eligible in the CHHiP trial so that the high-risk group remained at 12%. In contrast, the present study comprised 51% of the high-risk group. Despite such differences, the CHHiP trial and the present study demonstrated similar tendencies.

There are few reports of the long-term results of HIMRT using TomoTherapy. Kong et al. reported incidences of acute and late toxicities after HIMRT for prostate cancer using TomoTherapy [30]. Grades 0, 1, 2, and 3 or more late genitourinary toxicities were 82.0%, 14.0%, 4.0% and 0.0%, respectively, and those of late gastrointestinal toxicity were 18.0%, 56.0%, 26.0% and 0.0% respectively. However, the treatment outcome was not mentioned in the study.

Reports of HIMRT for prostate cancer from Japan are limited [20–22]; however, some studies reported three-dimensional conformal radiotherapy for prostate cancer. Akimoto et al. reported late toxicity of a total dose of 69 Gy in 3 Gy fractions in 3 days a week using three-dimensional conformal radiotherapy [20]. In the study, rectal bleeding of grade 2 or worse was observed in 25% patients. According to the report from Akimoto et al. [20], to reduce the rectal toxicity, the treatment schedule in the present study was set at the total dose of 66 Gy in 22 fractions for 3 days a week. Akimoto et al. also reported the clinical outcome of radiotherapy using a total dose of 69 Gy in 3 Gy fraction three times in a week for localized hormone-refractory prostate cancer, 3-year and 5-year cause-specific survival rates of 94% and 87%, respectively, and 3-year and 5-year clinical relapse-free survival rates of 78% and 56%, respectively [21]. Compared with the two studies from Japan, it was suggested that late rectal toxicity was reduced without impairing the effect of the treatment by using IMRT in the present study.

There are various treatment schedules in hypofractionated radiotherapy for prostate cancer, and the optimal radiation schedule or dose for the curative treatment of prostate cancer has not been established. Therefore, further long-term observation and clinical trials are required to determine the optimal dosage or treatment schedule. However, favorable clinical outcomes of hypofractionated radiotherapy for prostate cancer have been reported. It appears that HIMRT for prostate cancer is increasingly promoted in the future. In recent studies of the hypofractionated radiotherapy for prostate cancer, treatments were predominately performed five times a week. However, the treatment schedule in the present study, with a total dose of 66 Gy in 22 fractions in 3 days/week, is considered one of the options of HIMRT for prostate cancer.

## Conclusions

Long-term analysis of effectiveness and toxicities for patients with localized or locally advanced prostate cancer treated with HIMRT using TomoTherapy has been reported in this study. This study demonstrated that HIMRT using TomoTherapy for prostate cancer has a favorable outcome with tolerable toxicity.

## Abbreviations

ADT: androgen deprivation therapy
CTV: clinical target volume
IMRT: intensity-modulated radiotherapy
HIMRT: hypofractionated intensity-modulated radiotherapy
PSA: prostate-specific antigen
PTV: planning target volume
